# Reducing energy availability in male endurance athletes: a randomized trial with a three-step energy reduction

**DOI:** 10.1080/15502783.2022.2065111

**Published:** 2022-05-25

**Authors:** Iva Jurov, Nicola Keay, Samo Rauter

**Affiliations:** a Faculty of Sport, University of Ljubljana, Slovenia; b Department of Sport and Exercise Sciences, Durham University, Durham, UK

**Keywords:** Estimating energy expenditure, physical activity, exercise testing

## Abstract

**Background:**

Low energy availability (EA) can be detrimental for athlete health. Currently, it is not known what the threshold for low EA in men is, and what effects it may have on performance.

**Methods:**

This study was set to determine potential effects of low EA by modulating male participants’ exercise energy expenditure and controlling energy intake and consequently manipulating EA in three progressive stages (reducing EA by 25%%, and 75 %). Performance was measured with three specific tests for explosive power, endurance, and agility. Blood was drawn, resting energy expenditure was monitored and two questionnaires were repetitively used to address any changes in eating behaviors and well-being.

**Results:**

Repeated measured design showed poorer performance (power output 391.82 ± 29.60 vs. 402.5 ± 40.03 W, *p* = 0.001; relative power output 5.53 ± 0.47 vs. 5.60 ± 0.47 W/kg, *p* = 0.018; explosive power 0.28 ± 0.04 vs. 0.32 ± 0.05 m, *p* = 0.0001, lactate concentration 7.59 ± 2.29 vs 10.80 ± 2.46 mmol/L, *p* = 0.001). the quartile range for testosterone was lower (2.33 ± 1.08 vs. 2.67 ± 0.78, *p* = 0.026) and there was a tendency for lower *triiodothyronine* (4.15 ± 0.61 vs. 4.46 ± 0.54 pmol/L, *p* = 0.072). Eating behaviors and well-being were worse (46.64 ± 7.55 vs. 24.58 ± 7.13, *p* = 0.011 and 15.18 ± 2.44 vs. 17.83 ± 3.54, *p* = 0.002). The intervention also resulted in lower body fat (8.44 ± 3.15 vs. 10.2 ± 2.5%, *p* = 0.013).

**Conclusions:**

Analysis showed that most of the negative effects occurred in the range of 9–25 kCal·kg∙FFM·d^−1^. This is the range where we suggest a threshold for LEA in men could be. Reducing EA impaired explosive power first, then endurance. It was associated with a reduction in testosterone, *triiodothyronine* and there was a tendency for reduced IGF-1, but hormones were more resilient to changes in EA. Psychological assessment of eating behaviors and well-being proved to be very useful, whereas monitoring resting energy expenditure did not.

## 1. Introduction

Relative energy deficiency syndrome in sport (RED-S) includes but is not limited to effects on health, performance and well-being [1]. Impairments of health can occur when physiological systems are affected. Potential negative performance effects can be caused both by altered health or well-being (mental health or psychological function). Male athlete triad is another definition used almost interchangeably with RED-S [2]. At this time, there is no data to suggest what is a critical duration of low energy availability (LEA) that will result in clinical symptoms of RED-S. What is more, the threshold of LEA, being energy availability (EA) < 30 kCal·kg∙FFM·d^−1^ [3] established in early studies in women is now in doubt. It is not certain that there is a clear cutoff EA value below which adverse consequences of insufficient EA occur [4–6]. It has already been suggested a range might more appropriately describe this phenomenon – on one side of the range is sufficient EA supporting well-being and on the other is insufficient EA, resulting in RED-S [7]. Theoretically, the greater the reduction in EA, the greater the probability becomes of negative impact on health and possibly performance. In addition, current research does not provide a clear threshold for LEA in males [8]. A LEA value of between 15 and 30 kCal·kg∙FFM·d^−1^ has been suggested [9,10] and is probable that males are indeed less prone to negative health consequences of LEA that female**s** [11], so the probability is that the threshold is lower [12].

This study aimed to reduce EA in well-trained and elite male endurance athletes in three progressive stages. The aim was to determine a threshold for LEA in men, confirmed by changes in parameters that change significantly when EA is reduced. Health, performance and well-being were monitored while reduced EA was achieved by increasing exercise energy expenditure (EEE) and controlling energy intake (EI).

## 2. Methods

### Ethics, consent, and permissions

National medical ethical approval was acquired before the start of the study (No. 0120-202/2020/5).

Eighteen healthy subjects were invited to participate in the study through national cycling and triathlon organizations, professional cycling team’s coaches. The information was also disseminated through faculty’s laboratory, where national best endurance athletes regularly perform various testings. Subjects were informed of all procedures and were selected based on inclusion criteria, high motivation and compliance. Participants were selected based upon the criteria, as per the methods of Jurov et al. [13] Additionally, participants were assessed for VO2max and were included in the study if their values were between 55.0 and 64.9 mL/kg/min (performance level 3 or more) of De Pauw et al. [14] [15]. The details of stages 1 (pending publication) and 2 [13] are not included in this paper due to overwhelming data that cannot be included in a single paper and can be found elsewhere. In this paper, we present the final analysis of the project consisting of three EA reduction phases and the determination of the threshold for LEA.

### Study design

All males set to complete the same protocol consisting of four phases. This research was set as a randomized trial. EA was reduced in trained, well-trained, and elite male endurance athletes (cyclists and triathletes) in three progressive stages assigned to participants in a random order. First, athletes were assessed in stage 0 to monitor their baseline values and confirm inclusion criteria. In addition, in stage 0 we measured their energy intake (EI) and exercise energy expenditure (EEE). Subjects had to by complete dietary diaries to calculate EI and EEE was monitored during all training units using wearable heart rate monitors (Polar V800, Polar Electro, Kempele, Finland). EA was calculated as EA = (EI-EEE)/FFM in normal living conditions [13]. Then, participants’ EA was reduced by 25% (stage 1), 50% (stage 2), and 75% (stage 3), in these three steps. The stages were assigned to athletes in random order and there was a 1-month wash-out period between each of them to ensure all parameters return to baseline values. The duration of the wash-out period was chosen based on a study by Papageorgiou et al. looking into reduced EA effects on bone metabolism [16]. We confirmed that participants reached the baseline values by repeating measurements on day 1 of each of the stages. The parameters measured were resting energy expenditure (REE), body composition, blood values, and two questionnaires. However, if an individual did not reach baseline values in this time, they had to start the next stage after additional 2 weeks or even later when the values returned to normal, which is blood values within the reference range and no more than 10% difference in REE, questionnaires and body composition results compared to baseline values

The results of the intervention in stages 1–3 were compared to results from stage 0. In this way, each athlete acted as a control in stage 0 and as participant in stages 1–3.

**Figure 1.**
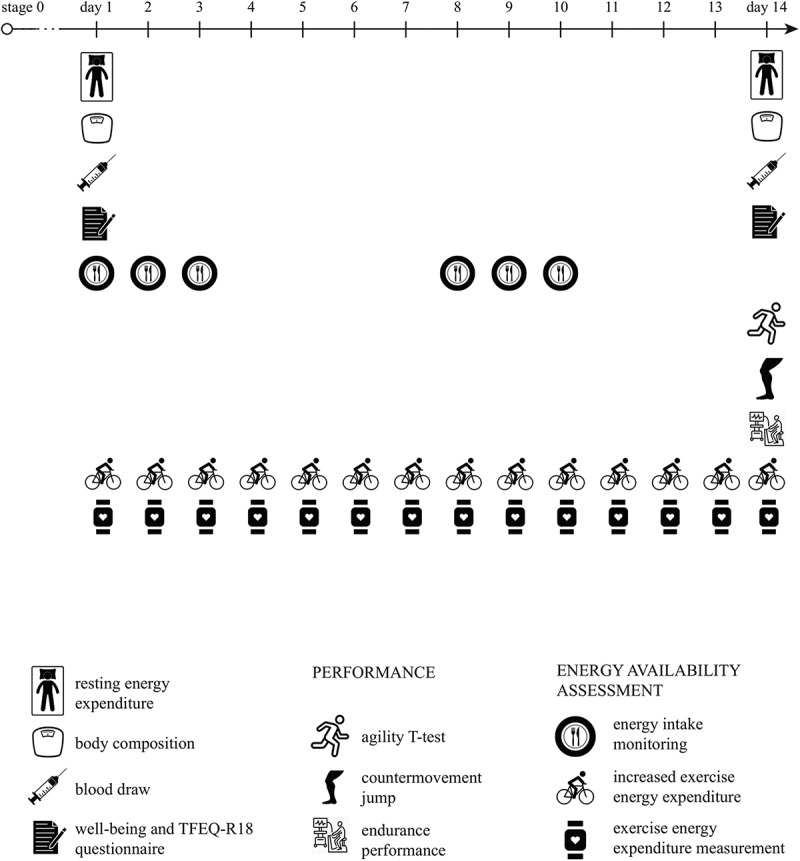
Stages 1-3 had the same 14-day timeline: measurements of health, REE and well-being were performed at the beginning and the end of each stage. In addition, three sport-specific performance tests were performed at the end of the stages.

In stages 1–3, EA was reduced (by 25%, 50%, and 75%) in all participants individually by increasing their EEE with cycling or running (Supplementary file 1). Subjects exercised every day for a specific time period adjusted for their EA reduction needs. They ran or cycled at HR between 70% and 80% of their maximal HR and the duration of their daily training was calculated for them in advance. Training time was calculated for every day and athletes were given the training schedule (with duration and intensity). Every day we checked their EEE by downloading their data from HR monitors and thus carefully controlled their EEE, which was distributed equally across the 14-day period (meaning they had to exercise for the same amount of time and kCal every day). Their EI remained the same as in stage 0. This was achieved by athletes repeating their dietary intake from stage 0 (they ate and drank the same amounts of the same meals with the exception of water and salt intake). They had to repeat their EI by following their eating schedule and this was controlled by repeating the diary log two times for 3 days in the 14 days (on day 1, 2, 3, 8, 9, and 10) (Figure 1). They did not repeat their dietary logs for the entire 14-day period to reduce the burden of reporting. In this way, their macronutrient intake ratio and EI remained identical as in stage 0. This enabled a new, lower EA. EEE was measured through all 14 days. On the 14th day, blood was be drawn, REE and body composition were measured, performance was assessed and The Three Factor Eating Questionnaire (TFEQ-R18) and Well-being questionnaires were completed.

EI measurement

EI was measured by completing dietary diaries for 7 consecutive days during stage 0 as per Capling et al. [17]. All participants received detailed information on how to complete the diary and how to weight the food or measure its quantity with the help of cups and other measuring tools. They were asked to provide photographic evidence on all food and liquid ingested in that time. EI data was analyzed with Foodworks 9 Professional Edition (version 9.0.3973, Xyrix Software, Australia).

EEE measurement

EEE was measured by wearing HR monitors during exercise (Polar V800, Polar Electro, Kempele, Finland). Body composition (FFM) was assessed with bioelectrical impedance (Biospace InBody 720, Seul, South Korea). Prior to body composition measurement, participants received instructions how to be adequately hydrated to enable precise measurement of FFM.

Assessing health

In the event that any participant experienced significantly reduced values of blood parameters indicating detrimental effects on health (−20% of baseline value), their protocol was terminated and they did not continue with the research.

For assessing health, venous blood was drawn and the following parameters were measured: Complete blood count, ferritin, serum Fe, triiodothyronine (T3), TSH, morning testosterone, fasting insulin, IGF-1, 9am cortisol. All blood tests were done at 9.00 a.m. in a fasted state.

Metabolic state was assessed by measured REE (mREE) and predicted REE (pREE), which is based on the Weir equation [18,19]. The mREE/pREE ratio was calculated. REE was measured with indirect calorimetry (V2 mask (Hans Rudolph, USA), K5). The measurement was performed in a thermoneutral environment, in silence, between 6.00 and 9.00 a.m., after 12 hours of fasting [20]. It lasted 30 min and last 20 min were used for REE measurement [21]. During every measurement RQ was monitored since measures under 0.70 or above 1 suggest protocol violations or inaccurate gas measurement [21].

Assessing performance

To test performance, three different tests were chosen to assess vertical jump height (explosive power of lower extremities), motor task execution time (agility), and maximal aerobic capacity (aerobic endurance).

Performance tests were performed in this exact order for each participant following the general warm up consisting of 2 min of cycling on a stationary bike at a 70 revolutions per minute at approximately 1.5 W/kg. Then they performed arm, hip, knee and ankle mobility exercises (10 reps each); dynamic stretches of hip flexors, knee extensors, knee flexors, and ankle extensors (10 reps each); and heel raise, squat, and crunch resistance exercises (10 reps each).

### Explosive power

Countermovement jump test was performed using a bilateral force plate system (Type 9260AA, Kistler Instrumente AG, Winterthur, Switzerland) with Kistler MARS software (S2P Ltd., Ljubljana, Slovenia) to acquire ground reaction force. Each subject performed three to five maximal counter movement jumps before the testing. For countermovement jump data were sampled at 1000 Hz, filtered using a moving average filter with 50-ms window and analyzed using the built-in module for countermovement jump. Test execution was supervised from the experienced researcher to improve proficiency in jumping technique [22]. Before each jump, participants were instructed to stand up straight and still on the center of the force plate with their hands akimbo. This hand position remained the same during the entire movement. From this position, participants initiated a fast downward movement until a crouching position with a knee angle of about 90°, followed by a jump for maximal height as quickly and explosively as possible. Three valid trials were performed with one-minute recovery period. The main outcome measure was countermovement jump height in centimeters that was calculated from the maximum velocity [23].

### Agility

To asses motor task execution time, validated modified agility *T*_test_ (called *T*_test_ in further text) was used, as described by Haj-Sassi et al. [24]. The *T*_test_ was performed after 5 minutes rest from vertical jump testing without additional warm up. Each participant had three trials (each trail consisting of two sprints separated by 30 seconds rest). The rest between trials was 90 seconds. Cones were placed in a T-shape layout, 5 and 2.5 m apart, respectively. Subjects were instructed to sprint and change direction as fast as possible. They began with both feet 0.3 m behind the starting line (A). At their own discretion subject sprinted forward to cone B and touched the base of it with the right hand. Facing forward and without crossing feet, they shuffled to the left to cone C and touched its base with the left hand. Subjects then shuffled to the right to cone D and touched its base with the right hand. They shuffled back to the left to cone B and touched its base. Finally, subjects ran backward as quickly as possible to cross the finish line (cone A) ending the first sprint. The second sprint started after 30 seconds rest. Three such trials separated by 90 seconds rest were performed, all with verbal encouragement. The subject who crossed one foot in to each direction (firstly starting with shuffling to the left) front of the other, failed to touch the base of the cone or failed to face forward throughout, had to repeat the test. The time to complete each repetition was measured using one pair of the electronic timing system sensors (Witty Timing System, Microgate, Bolzano) mounted on tripods. They were set approximately 0.75 m above the floor positioned 2 m apart facing each other on either side of the starting line (A). The time of best repetition (seconds) and three repetition average were used in further analysis.

### Aerobic endurance

Endurance was measured with the incremental test to exhaustion. Heart rate, ventilatory, and gas data were collected during the incremental test with metabolic cart (K5, Cosmed, Albano Laziale, Rome, Italy). All measurements were performed in the laboratory. For measuring VO_2_max, the following procedure was performed. After a 15-minute warm-up on bicycle set up on a cycle ergometer (Cyclus 2, Germany), workload constantly increased until volitional exhaustion (100 W + 20 W every minute). Ambient temperature in the laboratory was 21°C. Lactate was analyzed in capillary blood drop from the earlobe. Samples were obtained at rest before any physical activities, at the end of the test and 5 minutes after the test. Lactate was analyzed with the blood lactate analyzer Biosen C_Line – EKF Diagnostics, Germany.

Assessing well-being

TFEQ-R18 was used to detect early changes in eating behaviors. The questionnaire was also used as a tool for detecting LEA as previously shown in the literature. Well-being was assessed by a simple questionnaire as as per the methods of Jurov et al. [13]. The original well-being questionnaire, as recommended by Hooper and Mackinnon [25], was modified by adding a question about the frequency of morning erection. This has previously been used on professional rugby players [26].

Statistical analysis

For statistical analysis, SPSS was used with a significance level of <0.05 (version 25.0, IBM SPSS Statistics, Chicago, Illinois, USA). Repeated measures ANOVA was used to detect any differences in health, performance and metabolic factors in stages 1, 2, and 3. Descriptive statistics was used to present anthropological factors, age, energy parameters, blood values, performance and psychological evaluation in all stages. For determining the threshold for LEA, we used the same method as Loucks & Thuma [3] in women: we compared the shapes of incremental responses of blood, performance and psychological parameters based on changes of EA, visually and statistically. For the purpose of threshold determination, we used only parameters that showed statistically significant dependency with EA based on the repeated measured design. In addition, dependent *T*-test was used to compare differences in health, performance, metabolic factors, and psychological evaluation in stages 1, 2, and 3. Minimal sample size needed for this study (*N* = 12) was calculated with the G*power 3.1.9.2 software, assuming expected effect size 0.47 as reported in previous studies [27], with type I(a) error = 0.05 and 30% dropout of participants.

## 3. Results

All 12 athletes completed stage 0, 1, and 2. Stage 3 was too severe for everybody to complete the predicted 14 days. Eleven athletes could manage 10 or more days of 75% EA reduction. The severity of this intervention and reasons for not completing all 14 days are summarized in Supplementary file 2.

The main effects of stage 1, 2, and 3 are represented in Table 1.

**Table 1.** Summary of the main findings in three-stage EA reduction phases

	25% EA reduction (14 days), stage 1	50% EA reduction (14 days), stage 2	75% EA reduction (10 days), stage 3
Mean EA (kCal·kg∙FFM·d^−1^)	22.4 ± 6.3	17.3 ± 5.0	8.82 ± 3.33
Correlations to EA	Increased cognitive restriction (*r = 0.528, p* = 0.039)	Hemoglobin (*r* = −0.557, *p* = 0.30) and testosterone (*r* = −0.532, *p* = 0.037), anaerobic threshold (*r* = −0.597, *p* = 0.02) and respiratory compensation point (*r* = −0.575, *p* = 0.025)	A trend for the lower TSH (*p* = 0.079), higher insulin (*p* = 0.072)
Significant changes in body composition		Lower body fat percent (*t* [12] = 3.36, *p* = 0.006)	Reduced body mass (*t* [11] = 5.19, *p* = 0.0001, *r* = 0.85), body fat (*t* [11] = 2.41, *p* = 0.037, *r* = 0.54) and a tendency for lower FFM (*t* [11] = 3.03, *p* = 0.069, *r* = 0.54)
Blood parameters, that changed significantly	Hemoglobin (*t* [12] = 2.65, *p* = 0.022), a tendency for lower iron (*p* = 0.066) and IGF-1 (*p* = 0.077)	A tendency for reduced hemoglobin (*p* = 0.100) and IGF-1 (*p* = 0.065)	Reduced T3 values (*t* [11] = 2.65, *p* = 0.024, *r* = 0.64) and a tendency for lower testosterone (*t* [11] = 1.84, *p* = 0.095)
Performance parameters, that changed significantly	Reduced explosive power (*t* [12] = 4.57, *p* = 0.001), altered lactate metabolism (*t* [12] = 2.84, *p* = 0.016)	Reduced power output (*t* [12] = 2.69, *p* = 0.021) and relative power output (*t* [12] = 2.34, *p* = 0.036), reduced explosive power (*t* [12] = 6.41, *p* = 0.0001), altered lactate metabolism (*p* = 0.001)	Reduced power output (*t* [11] = 2.30, *p* = 0.044), explosive power (*t* [11] = 5.93, *p* = 0.0001) and significantly lower lactate values (*p* = 0.0001)
Psychological parameters, that changed significantly	Poorer well-being (*t* [12] = 2.385, *p* = 0.036)	Ppoorer well-being (*t* [12] = 4.11, *p* = 0.002) and eating behaviors (*t* [12] = −2.71, *p* = 0.020)	Poorer well-being (*t* [11] = 5.13, *p* = 0.0001), eating behaviors (*t* [11] = −4.28, *p* = 0.002) and increased cognitive restriction (*t* [11] = −2.56, *p* = 0.028)
Number of subjects with mREE/pREE<0.9	3/12	3/12	5/12

The results of the repeated measured design showed that the following parameters were significantly affected after EA was reduced. Performance was poorer (PO, *p* = 0.001; RPO, *p* = 0.018; CMJ, *p* = 0.0001; La_max_, *p* = 0.001; La_5min_, *p* = 0.001). The quartile range for testosterone (reference range for testosterone being divided into four quartiles to detect first changes in testosterone values) was lower (*p* = 0.026), there was a tendency for lower T3 (*p* = 0.072). TFEQ-R18, CR and well-being were worse (*p* = 0.011, *p* = 0.082, and p = 0.002). The reduction of EA resulted in lower body fat (*p* = 0.013). The assumption of sphericity was not violated in any of parameters. The effects size measures for focused comparisons between interventions can be found in the Supplementary file 1 (Table 2), as well as details on stage 3 in Tables 3–8. More details on data obtained at stage 1 (to be published) and stage 2 [15] can be found elsewhere.

**Figure 2.**
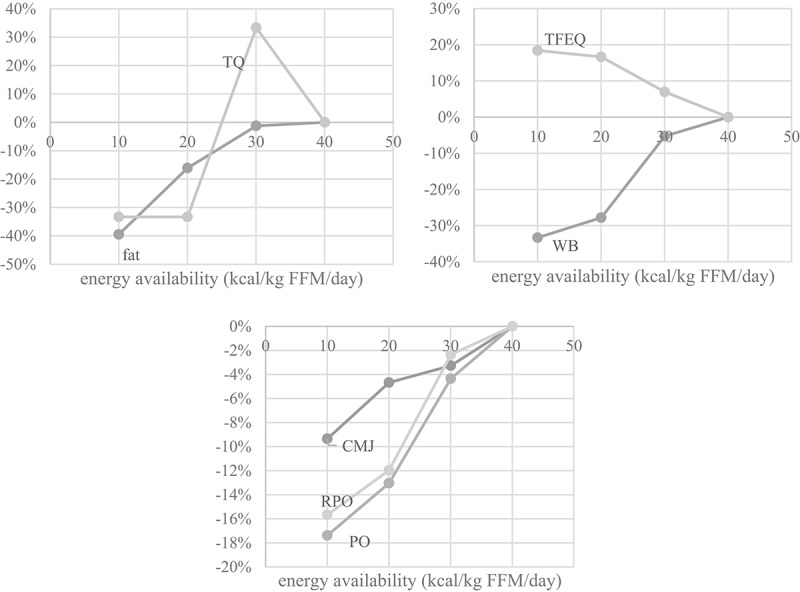
Effects of reduced EA on body fat % (fat) and testosterone quartile reference range (TQ, top left), on mental state as assessed by the Three Factor Eating Questionnaire (TFEQ-R18) and Well-being questionnaire (WB, top right) and on performance as measured by the countermovement jump (CMJ), relative power output (RPO) and power output (PO, bottom) in a representative athlete. The differences are expressed in percent relative to values acquired before the intervention.

Figure 2 shows body composition, blood, performance, and psychological parameters profile in the three stages of reduced EA (−25%, −50%, and −75%) in a representative athlete. In Figure 3, mean differences of parameters for all subjects are represented for all stages. Only the parameters that showed significant effects by EA in the repeated measured design were used.

**Figure 3.**
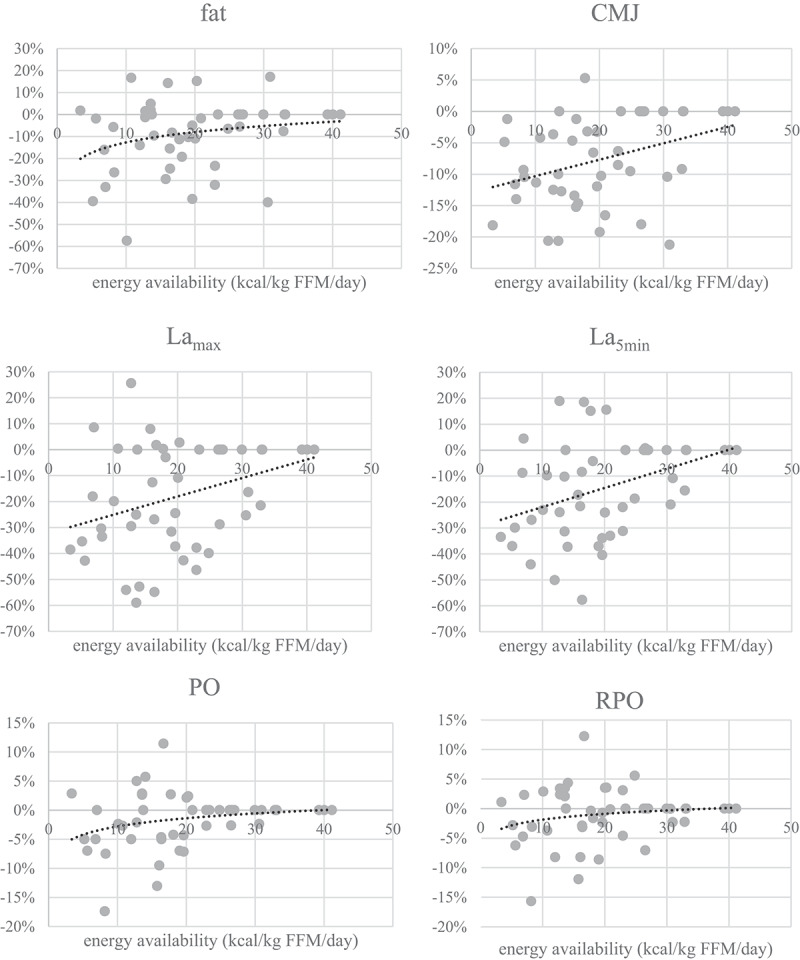
Changes of anthropometric, performance, psychological and blood parameters at different EA values. All changes are expressed relative to values before the intervention in percent (fat=body fat percent, CMJ=countermovement jump, PO=power output, RPO=relative power output, La_max_=lactate concentration at the end of the incremental test, La_5min_=lactate concentration 5 minutes after the end ofthe incremental test, WB=well-being questionnaire, TFEQ-R18=the Three Factor Eating Questionnaire,TQ=testosterone reference range quartile).

**Figure 3.**
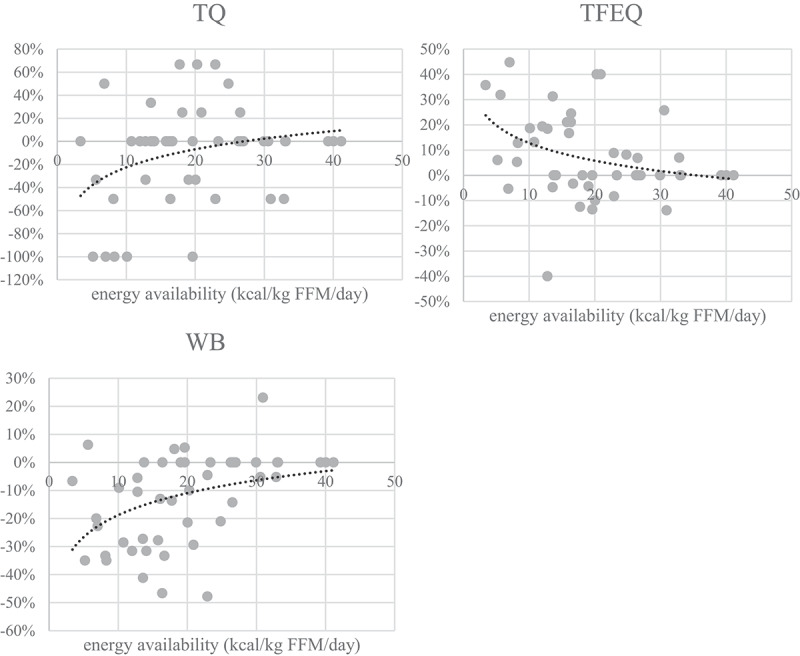
Changes of anthropometric, performance, psychological and blood parameters at different EA values. All changes are expressed relative to values before the intervention in percent (fat=body fat percent, CMJ=countermovement jump, PO=power output, RPO=relative power output, La_max_=lactate concentration at the end of the incremental test, La_5min_=lactate concentration 5 minutes after the end ofthe incremental test, WB=well-being questionnaire, TFEQ-R18=the Three Factor Eating Questionnaire,TQ=testosterone reference range quartile).

**Figure 4.**
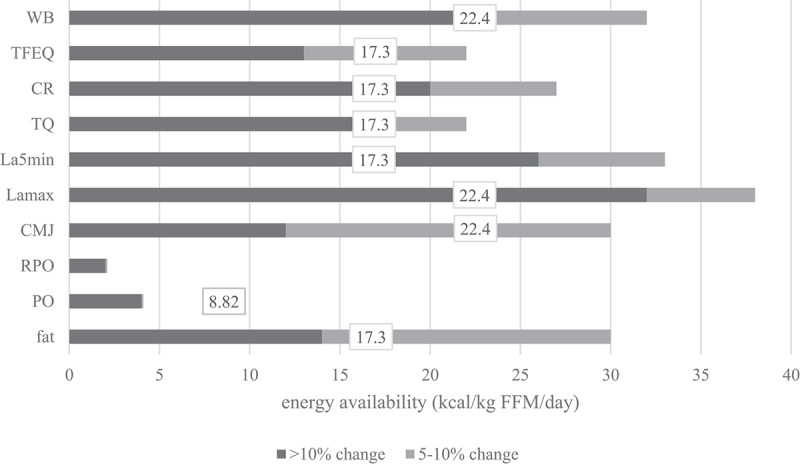
Mean EA values when differences in measured parameters were >10% (black), 5-10%(grey) and mean EA value when T-test firstly showed a significant difference in parameter after EA reduction (number in white square). Determining the threshold for LEA in men is most probably a spectre between observed parameters as LEA affects health, performance and mental state and all parameters are not affected at the same EA absolute value. Also, there are clearly individual differences in resilience to reduced EA in 14 days. This chart suggests the spectre could be between 2and 32 (when mean difference is >10%), with mean value of 17 kcal·kg∙FFM·d-1. In the stage 2 (EA=17.3±5.0 kcal·kg∙FFM·d-1) we also observed statistically poorer values in the most of the observed parameters (fat=body fat percent, CMJ=countermovement jump, PO=power output, RPO=relativepower output, La_max_=lactate concentration at the end of the incremental test, La_5min_=lactate concentration 5 minutes after the end of the incremental test, WB=well-being questionnaire, TFEQR18=the Three Factor Eating Questionnaire, TQ=testosterone reference range quartile).

Visual representation of the threshold determination is shown in Figure 4. To determine at which value health, performance and mental state change, three approaches were used: mean difference in parameter is >5%, mean difference is >10% and EA mean value when *T*-test showed significant differences (in the stage with the least EA reduction, i.e. when the change firstly appeared).

## 4. DISCUSSION

The aims of this study were to assess if reduced EA has any negative effects on health, performance, or well-being and to determine the threshold for LEA in a sample of trained and well-trained male endurance athletes. The four main findings were: (1) There is no threshold for LEA in our sample but rather a range, where health, performance, and psychological factors alter at different EA values; (2) this range starts at significantly lower values than in female athletes; (3) a future assessment tool for reduced EA might benefit from assessing well-being and eating behaviors with a combination of measuring testosterone and IGF-1 changes from an athlete’s baseline values; (4) it seems that mental state and performance are affected before health.

This is the first study with a three-step approach to reducing EA in male athletes. In females, the threshold was determined with a similar approach as in this study, but only one parameter was used – luteinizing hormone pulsatility [3]. We believe that using more parameters as in this stage is in line with conclusions of the IOC consensus group, which is that LEA affects many aspects of health and performance, not only the endocrine system [1]. In accordance with evidence in females [28], our study does not support the use of an absolute EA threshold in males. Based on our results of reduced EA over 14 days, we propose a range with a mean value of 17 kCal·kg∙FFM·d^−1^. Since EA reduction usually lasts more than 14 days in real time, rather than using the range of 2–32 as shown with 10 parameters used in our study, we propose using a narrower range of 9–25 kCal·kg∙FFM·d^−1^. There are also some other studies assessing effects of reduced EA on males in the proposed range. Papageorgiou et al. found that 5 days of 15 kCal·kg∙FFM·d^−1^ in physically active men was not enough to show decreased bone formation and increased bone resorption [16]. Koehler et al. [9] also observed effects of 15 kCal·kg∙FFM·d^−1^ lasting four days in exercising men (*n* = 6) and detected reductions in leptin and insulin, whereas ghrelin, T3, testosterone and IGF-1 did not change significantly. Heikura et al. [29] looked into 8 days of multiple single-day cycling races interrupted by recovery days. They observed that cyclist having severely reduced EA during races (<10 kCal·kg∙FFM·d^−1^; *n* = 2) experienced a trend toward decreased testosterone and IGF-1 (−25%). More studies should be performed to draw any relevant conclusions and to explore if there are any differences in sport disciplines and level of fitness.

### Well-being and performance are affected before hormonal changes

Clearly, the more severe EA reduction, the more parameters will be affected faster, but we can report some differences in the timing of these effects. First, well-being, explosive power and lactate metabolism are affected. Then, at 50% EA reduction (EA = 17.3 ± 5.0), eating behaviors are at risk and body fat is significantly reduced after 14 days. Lastly, at 75% EA reduction, power output is significantly lower even after 10 days. Testosterone showed individual responses to EA reduction, but as shown by repeated measures design, reducing EA impacts the testosterone quartile reference range which dropped rapidly in 14 days. Our results add to the findings of Koehler et al. who also failed to show reduction of absolute testosterone values when EA was 15 kCal·kg∙FFM·d^−1^ for 5 days [9]. They also found a tendency for lower IGF-1. A trend for decreased testosterone and IGF-1 was also found in an alternate-day low EA (<10 kCal·kg∙FFM·d^−1^) in 8 days [29]. It seems that 14 days as used in our study design is also not sufficient to show significant changes without a bigger sample size. It does, however, suggest, that monitoring IGF-1 and testosterone might be informative when looking for indications of EA.

We would like to emphasize that we observed great individual variability in response to the severely reduced EA. As the 75% reduction phase lasted only 10 days, there were not many statistically different values in blood parameters, but we observed nine critically altered values that fell outside the reference range. This adds to the evidence for a continuum between optimal EA and critically low EA, where individuals react with different degrees of resilience. This is also a reason why we suggest that there is no absolute threshold for LEA.

The Well-being questionnaire also assessed functional physiological testosterone effect (morning erection). Libido is suspected to be reduced in intensive endurance training [30], but there is no evidence gathered from controlled research [31]. Our study design did not show differences in functional testosterone effect, despite controlled and strictly monitored conditions. Since we detected changes in measured testosterone, a drop in testosterone quartile reference range and correlation in stage 2, we suspect we failed to detect changes in functional effect due to unwillingness to share private information. A rephrased question might be useful in a future questionnaire looking for reduced EA in male athletes. This might be done by asking them if they felt their desire for sexual interactions was decreased instead of asking directly about morning erections.

### Practical application

Based on these results it might be useful to use the Well-being questionnaire repetitively (every 14 days) to assess any negative effects of reduced EA. Eating behaviors should be assessed at least at changes in training blocks (every few months) and especially when body mass is regulated. To avoid suboptimal endurance performance, severe EA reduction must be avoided even in shorter training blocks of 10 days. In addition, to ensure optimal explosive power in lower limbs (in competition phase especially), EA should be optimal, as even a 25% reduction has a negative impact. Since athletes can experience reduced EA for a longer time than just 14 days, we suggest checking changes in testosterone, IGF-1, T3, iron and hemoglobin from baseline values. These could be helpful when assessing the risk for LEA without the tedious measurement of EA. We would, however, like to stress that these values might still be in reference range.

### Can measuring resting energy expenditure be useful?

Reducing EA in three steps did not elicit any significant changes in mREE/pREE as suggested by some authors [31]. This study failed to confirm that energy conservation as detected by mREE/pREE could be used for suboptimal EA assessment. This is consistent with findings that healthy men do not have lower mREE/pREE despite a more negative energy balance [19]. Future studies should aim to assess longer duration of EA on mREE/pREE. Our study design was set to measure mREE 12 or more hours after the last training unit. Since increased training load might have an impact in our measurements with the EPOC effect [32,33], we suggest future studies should measure mREE 24 or even 36 hours after the last training unit. Energy conservation could be affected by reduced EA, but using it as an assessment tool might not be so practical if 36 hours of inactivity need to pass before the measurement takes place.

### Future directions

Based on these results, it seems reasonable to do more research with a more moderate EA reduction (25–40%) over a longer time frame to understand how duration affects athlete’s status. The 50% and 75% reduction might not be sensible as athletes barely managed to stay on desired EEE levels in stage 2 and they were not able to finish more than 10 days in stage 3. It would also be beneficial to observe if any other sport modalities change if EA is reduced, like maximal power or reaction time. The diet of athletes, that on average consisted of 18% protein, 42% carbohydrate, and 35% fat, was not changed in macronutrient ratios when EA was reduced in this study. If EA would be reduced by maintaining protein intake and reducing only carbohydrate and fat, the observed effects could be different. We suggest dietary manipulation with EA measurement is a necessary next step to better understand the mechanisms behind LEA.

Another parameter that would enable direct comparison of the threshold for LEA in men to the threshold for women as determined by Loucks et al. [3] would be measuring the pulsatility of luteinizing hormone in men with reduced EA. However, this might not be possible for trained and elite athletes as part of research due to high burden on the participants when luteinizing hormone is measured. Our study design is particularly beneficial to this area of knowledge, because we selected athletes who compete or competed in the past nationally and internationally, are of high performance level and showed great compliance with the study regime. Lastly, it is imperative that a simpler assessment tool like a questionnaire is devised to detect suboptimal EA in male athletes early. Using the range proposed by this study might prove to be more useful than EA = 30 kCal·kg∙FFM·d^−1^ as used in women and men previously. We suggest that a too high and EA cutoff value in previous research for devising a questionnaire in men might be one of the reasons why inconclusive results were found and still no standardized simple questionnaire has been developed.

### Limitations

The authors would like to acknowledge that although all participants are trained endurance athletes, some of them fall into the well-trained and also elite group according to De Pauw et al. [14]. Finding sufficiently large sample size of only elite athletes is a challenge in such a research setting. This is why we used athletes from all three performance groups. To our knowledge, this is the biggest sample in a controlled setting for measuring EA in trained endurance male athletes and can thus provide some relevant conclusions for scientists, athletes, their coaches and other sports practitioners. Only this approach can enable further exploration of LEA effects on the male hypothalamic-pituitary-gonadal axis (Logue et al., 2020) and performance consequences.

The second limitation of this study is that the severity of LEA did not enable all of the athletes to complete the stage 3 in full duration (and one subject in stage 2). As explained in the discussion, this limitation also proves the reduction of EA was severe and that it indeed has negative impact on mental state, health and performance. Ideally, all participants would finish the whole 14 days in all stages, but this was not feasible due to our concern for their health. Lastly, dual energy X-ray absorptiometry (DXA) has been considered a gold standard for measuring FFM [34,35], but our study used BIA. This method requires subjects to be in euvolemic state – attending to good hydration prior to measurement, but is otherwise a more convenient method that can be repeated multiple times with no radiation (as is the case in DXA). Our participants were instructed to be well hydrated prior all body composition measurements, but we acknowledge that there might be some differences if DXA would be used. Our findings are still a great contribution to current knowledge and are in line with the literature that hypothesized severe EA as reached in stage 3 is not compatibe with optimal training process and striving for great results in endurance athletes.

## 5. Conclusions

This was the first systematic intervention study in male endurance athletes that reduced EA in three stages in random order. By measuring EA and using athletes as controls and intentionally reducing their EA, we were able to find differences in the outcome variables of health, performance, and well-being for the first time, without estimates or indirect assumptions about the presence of LEA. Analysis of all the data collected confirmed that our sample of male endurance athletes is more resilient to reduced EA than women. We can propose that a range exists for LEA in endurance male athletes that is lower than the proposed threshold in women. We suggest that a range of 9-25 kCal·kg∙FFM·d^−1^ could be used. These findings should be explored further in male athletes of other disciplines to evaluate if this range could be used for determining LEA males. Based on our results, we have highlighted the parameters that may prove most useful in developing tools to assess reduced EA in the future.

## Supplementary Material

Additional File 1

Additional File 2

## Data Availability

Data are available upon reasonable request

## List of abbreviations

[La]_5min_ – lactate concentration 5 minutes after the end of the test

[La]_max_ – lactate concentration at the end of the test

CMJ – countermovement jump

CR – cognitive restriction

EA – energy availability

EEE – exercise energy expenditure

EI – energy intake

FFM – fat free mass

IGF-1 – insulin like growth factor 1

LEA – Low Energy Availability

mREE – measured resting energy expenditure

PO – peak power output

pREE – predicted resting energy expenditure

RED-S – Relative Energy Deficiency in Sport syndrome

RPO – relative power output

T3 *–* triiodothyronine

TFEQ-R18 – Three Factor Eating Questionnaire

TSH – thyroid stimulating hormone

VO_2max_ – maximal oxygen uptake
