# Supplementary material for: Reducing energy availability in male endurance athletes: a randomized trial with a three-step energy reduction
Source: J Int Soc Sports Nutr. 2022 May 25;19(1):179–95. doi: 10.1080/15502783.2022.2065111 (PMC9261741; doi:10.1080/15502783.2022.2065111)
Supplement: Additional File 1 [file RSSN_A_2065111_SM7940.docx]

**Additional file 2**

In stage 3, the severity of EA reduction caused some athletes finish the intervention before the proposed 14 days (Table 2). Among hunger, empty energy reserves, inability to train at the same intensity levels as expected, the progressively lower HR levels were reported in 5/11 subjects.

Table 3: Subjective assessment of stage 3 from all 12 participants

| 1 | too tired to finish, hungry |
| --- | --- |
| 2 | heavy legs, inability to lift HR as usual, fatigue, hunger |
| 3 | very hungry, inability to perform well, empty energy reserves, can start training normally but then fails to continue almost immediately |
| 4 | very fatigued, low HR during training sessions, inability to raise HR, feels completely empty, poor sleep, heavy legs |
| 5 | inability to reach desired EEE levels, hungry |
| 6 | migraine, low energy levels, very easily irritated, sleepy during the day, then inability to fall asleep, lost appetite, is not hungry, very apathic in family setting |
| 7 | hard and heavy legs, irritable, increasing difficulty to concentrate and work, not extremely hungry, very hard to fall asleep, 1 panic attack - afraid that will not be able to finish |
| 8 | increasing fatigue and hunger |
| 9 | hungry, some mild chest pain during sleep, got used to the load in the last days but progressively hungry, irritated easily |
| 10 | empty energy stores, poor sleep, irritability, apathy, needed to take a break during training in the last few days. In the first days there was great hunger, then it almost disappeared and he couldn't eat a lot - felt full almost immediately. He also noticed progressively reduced HR after only few days. |
| 11 | very hungry, tired, headache 3 days, needed breaks to finish training, empty energy stores |
| 12 | very hungry, inability to raise HR, increasing fatigue in the second week, heavy legs, felt like he had no power, wasn't able to train in high intensity |

The 75% EA reduction in stage 3 resulted in changes in body composition (Table 4), blood parameters (Table 5), performance measures (Table 6), and in psychological evaluation (Table 7). Blood parameters correlations to EA are presented in Table 8.

Table 4: Differences in body composition at the beginning (pre) and at the end (post) of stage 3

|  | Mean | | Std. Deviation | | Std. Error Mean | | 95% CI Lower | | 95% CI Upper | t | | df | | Sig. (2-tailed) |
| --- | --- | --- | --- | --- | --- | --- | --- | --- | --- | --- | --- | --- | --- | --- |
| BM_pre-post (kg) | 2.11 | | 1.35 | | 0.41 | | 1.20 | | 3.01 | 5.189 | | 10 | | **0.0001*** |
| FFM_pre-post (kg) | 0.73 | | 1.19 | | 0.36 | | -0.07 | | 1.52 | 2.033 | | 10 | | 0.069 |
| FAT_pre-post (%) | 1.52% | 2.09% | | 0.63% | | 0.11% | | 2.92% | | 2.407 | 10 | | **0.037*** | |

*statistical significance set at p<.05

(BM=body mass, FFM=fat-free mass, FAT=fat mass)

Table 5: T-test comparing blood parameters before and after intervention in stage 3

| Marker | Reference range | Mean 3a | Mean 3b | Mean Difference | Std. Deviation | Std. Error Mean | 95% CI Lower | 95% CI Upper | t | df | sig. (2-tailed) |
| --- | --- | --- | --- | --- | --- | --- | --- | --- | --- | --- | --- |
| Haemoglobin (g/L) | 138 - 175 | 145.73 | 144.00 | 1.73 | 7.48 | 2.26 | -3.30 | 6.76 | 0.765 | 10 | 0.462 |
| S-Iron (μmol/L) | 5.8 - 34.5 | 24.13 | 25.800 | -1.67 | 11.55 | 3.48 | -9.43 | 6.08 | -0.481 | 10 | 0.641 |
| S-TSH (mIU/L) | 0.27 - 4.20 | 2.16 | 2.26 | -0.09 | 0.66 | 0.20 | -0.54 | 0.35 | -0.470 | 10 | 0.649 |
| S-T3 (pmol/L) | 3.1 - 6.8 | 4.64 | 4.15 | 0.48 | 0.61 | 0.18 | 0.08 | 0.89 | 2.648 | 10 | **0.024*** |
| S-Testosterone (nmol/L) | 8.64 - 29.0 | 19.33 | 14.84 | 3.49 | 6.29 | 1.90 | -0.73 | 7.72 | 1.844 | 10 | **0.095** |
| S-cortisol (nmol/L) | 166.0-507.0 | 456.52 | 427.91 | 28.61 | 60.72 | 18.31 | -12.18 | 69.40 | 1.563 | 10 | 0.149 |
| S-ferritin (μg/L) | 30 - 400 | 131.86 | 132.48 | -0.62 | 32.03 | 9.66 | -22.14 | 20.90 | -0.064 | 10 | 0.950 |
| Insulin (mE/L) | 2-29.1 | 2.61 | 2.627 | -0.02 | 1.21 | 0.36 | -0.83 | 0.79 | -0.050 | 10 | 0.961 |
| IGF-1 (μg/L) | 83.4-232.7 | 165.82 | 156.82 | 9.00 | 40.11 | 12.09 | -17.95 | 35.95 | 0.744 | 10 | 0.474 |
| IGF-1 SD |  | -0.38 | -0.619 | 0.234 | 0.852 | 0.257 | -0.34 | 0.807 | 0.912 | 10 | 0.383 |

*significance set at p<.05

(TSH=thyroid stimulating hormone, T3= triiodothyronine, IGF-1=insulin-like growth factor 1)

Table 6: Paired samples test of performance parameters in stage 3 and stage 0

|  | Mean | Std. Deviation | Std. Error Mean | 95% CI Lower | 95% CI Upper | t | df | Sig. (2-tailed) |
| --- | --- | --- | --- | --- | --- | --- | --- | --- |
| T-TEST_0 - 3 (s) | -0.02 | 0.44 | 0.13 | -0.32 | 0.28 | -0.136 | 10 | 0.895 |
| CMJ_0 - 3 (m) | 0.03 | 0.02 | 0.01 | 0.02 | 0.05 | 5.928 | 10 | **0.0001*** |
| VO_2max_ 0 - 3 (ml/min/kg) | 0.15 | 3.50 | 1.05 | -2.19 | 2.50 | .147 | 10 | 0.886 |
| PO_0 - 3 (W) | 17.27 | 24.94 | 7.52 | 0.52 | 34.03 | 2.297 | 10 | **0.044*** |
| RPO_0 - 3 (W/kg) | 0.15 | 0.35 | 0.10 | -0.08 | 0.39 | 1.481 | 10 | 0.170 |
| La_max__0 – 3 (mmol/l) | 2.99 | 2.15 | 0.65 | 1.55 | 4.44 | 4.608 | 10 | **0.001*** |
| [L_5min__0 – 3 (mmol/l) | 2.94 | 1.86 | 0.56 | 1.69 | 4.19 | 5.238 | 10 | **0.0001*** |

*statistical significance set at p<.05

(VO_2max_=maximal oxygen consumption, PO=peak power output, RPO=relative power output, AT=anaerobic threshold, RC=respiratory compensation point, La_max_=lactate concentration at the end of the test, La_5min_=lactate concentration 5 minutes after the end of the test, CMJ=countermovement jump)

Table 7: Differences in psychological evaluation before (pre) and after (post) intervention in stage 3

|  | Mean | Std. Deviation | Std. Error Mean | 95% CI Lower | 95% CI Upper | t | df | Sig. (2-tailed) |
| --- | --- | --- | --- | --- | --- | --- | --- | --- |
| WB_pre-post | 4.27 | 2.76 | 0.83 | 2.42 | 6.13 | 5.134 | 10 | **0.0001*** |
| TFEQ-R18_pre-post | -6.91 | 5.36 | 1.62 | -10.51 | -3.31 | -4.278 | 10 | **0.002*** |
| CR_pre-post | -3.55 | 4.59 | 1.38 | -6.63 | -0.46 | -2.562 | 10 | **0.028*** |

*statistical significance set at p<.05

(WB=well-being questionnaire, TFEQ-R18=three factor eating questionnaire, CR=cognitive restriction)

Table 8: Blood parameters correlations to energy availability (EA) at the end of stage 3

|  | | haemoglobin_3b | S-Iron_3b | S-TSH_3b | S-T3_3b | S-Testosterone_3b | S-cortisol_3b | S-feritin_3b | insulin_3b | IGF-1_3b | IGF-1 SD_3b |
| --- | --- | --- | --- | --- | --- | --- | --- | --- | --- | --- | --- |
| EA_3 real | **r** | -0.002 | -0.144 | 0.457 | 0.173 | 0.306 | 0.327 | 0.360 | -0.471 | -0.167 | 0.224 |
|  | **p** | 0.498 | 0.336 | **0.079** | 0.306 | 0.180 | 0.163 | 0.139 | **0.072** | 0.311 | 0.254 |
|  | **N** | 11 | 11 | 11 | 11 | 11 | 11 | 11 | 11 | 11 | 11 |

*. Correlation is significant at the 0.05 level (1-tailed).

(r=Pearson correlation, p=significance)

(TSH=thyroid stimulating hormone, T3= triiodothyronine, IGF-1=insulin-like growth factor 1)
