# Supplementary material for: Reducing energy availability in male endurance athletes: a randomized trial with a three-step energy reduction
Source: J Int Soc Sports Nutr. 2022 May 25;19(1):179–95. doi: 10.1080/15502783.2022.2065111 (PMC9261741; doi:10.1080/15502783.2022.2065111)
Supplement: Additional File 2 [file RSSN_A_2065111_SM7937.docx]

**Additional file 1**

EA for each stage was individually set based on values measured at stage 0. EA was reduced by 25% in stage 1, by 50% in stage 2 and by 75% in stage 3 (Figure 5).

*Figure 5:* Scatterplot of EA set for each stage

Average time for EEE in stage 0 (M=1172.78±419.61kcal/day) was 2 hours and 4 minutes (80.6% spent cycling, 9.3% running and 10.1% swimming). To reach the desired EA in stages 1-3, EEE values were calculated accordingly (Figure 6).


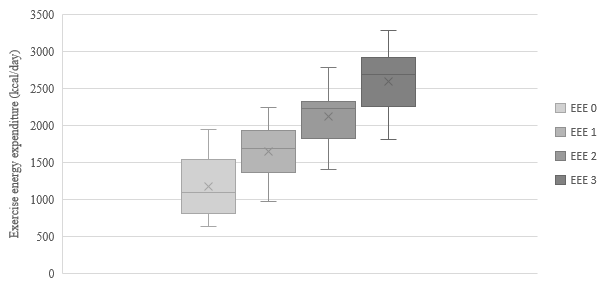


Figure 6: Calculated EEE values for determined EA in stages 1, 2 and 3

In stage 1, participants spent 15 hours 24 minutes cycling, 1 hour 41 minutes running and 45 minutes swimming on weekly average. Average daily training time was 2 hours 33 minutes per day. In stage 2, they were cycling 17 hours and 9 minutes, running 1 hour and 32 minutes and swimming 1 hour and 4 minutes per week. Average cumulative training time was 2 hours and 50 minutes per day. Finally, in stage 3 athletes spent 20 hours and 24 minutes cycling, 55 minutes running and 22 minutes swimming per week. On average, training time was 3 hours and 6 minutes per day.

Table 2: effect size (r) measures for focused comparisons between interventions of 25% (stage 1), 50% (stage 2) and 75% EA reduction (stage 3)

|  | r  (stage 1) | r  (stage 2) | r  (stage 3) |
| --- | --- | --- | --- |
| PO | 0.14 | **0.79** | 0.10 |
| RPO | 0.22 | **0.69** | 0.21 |
| CMJ | **0.80** | 0.10 | 0.12 |
| La_max_ | **0.62** | 0.41 | 0.01 |
| QT | 0.42 | 0.14 | **0.59** |
| T3 | 0.45 | 0.03 | **0.64** |
| TFEQ-R18 | **0.60** | **0.60** | 0.25 |
| WB | 0.41 | **0.70** | 0.23 |
| body fat | **0.53** | 0.41 | 0.10 |

(PO=power output, RPO=relative power output, CMJ=countermovement jump, La_max_=lactate concentration at the end of the incremental test, TQ=testosterone reference range quartile, T3=triiodothyronine, TFEQ-R18=the Three Factor Eating Questionnaire, WB=well-being questionnaire, fat=body fat percent).
